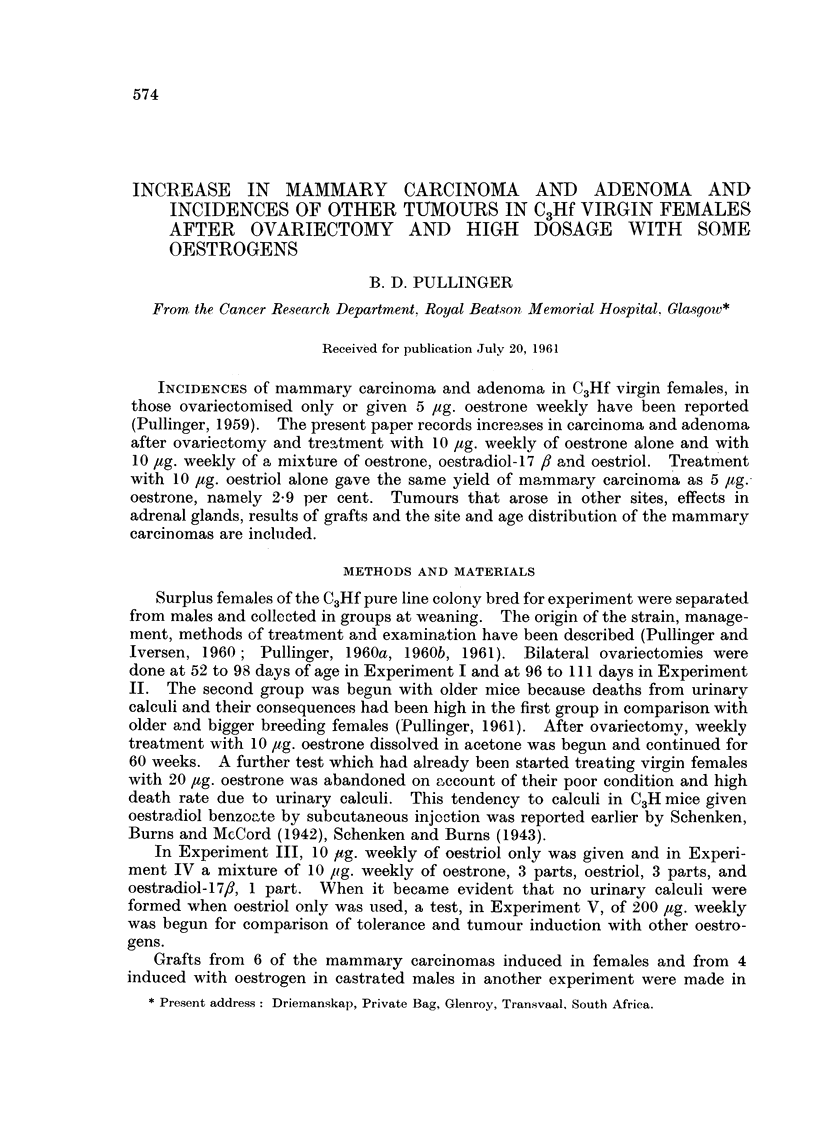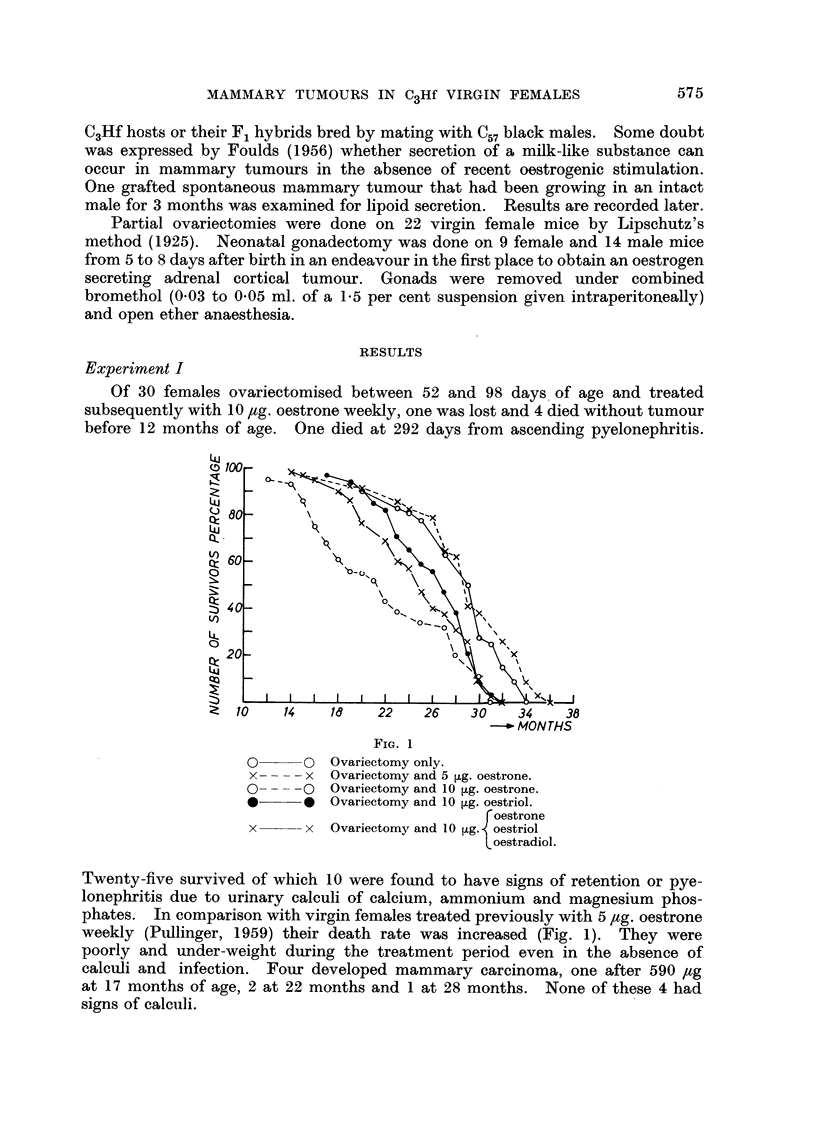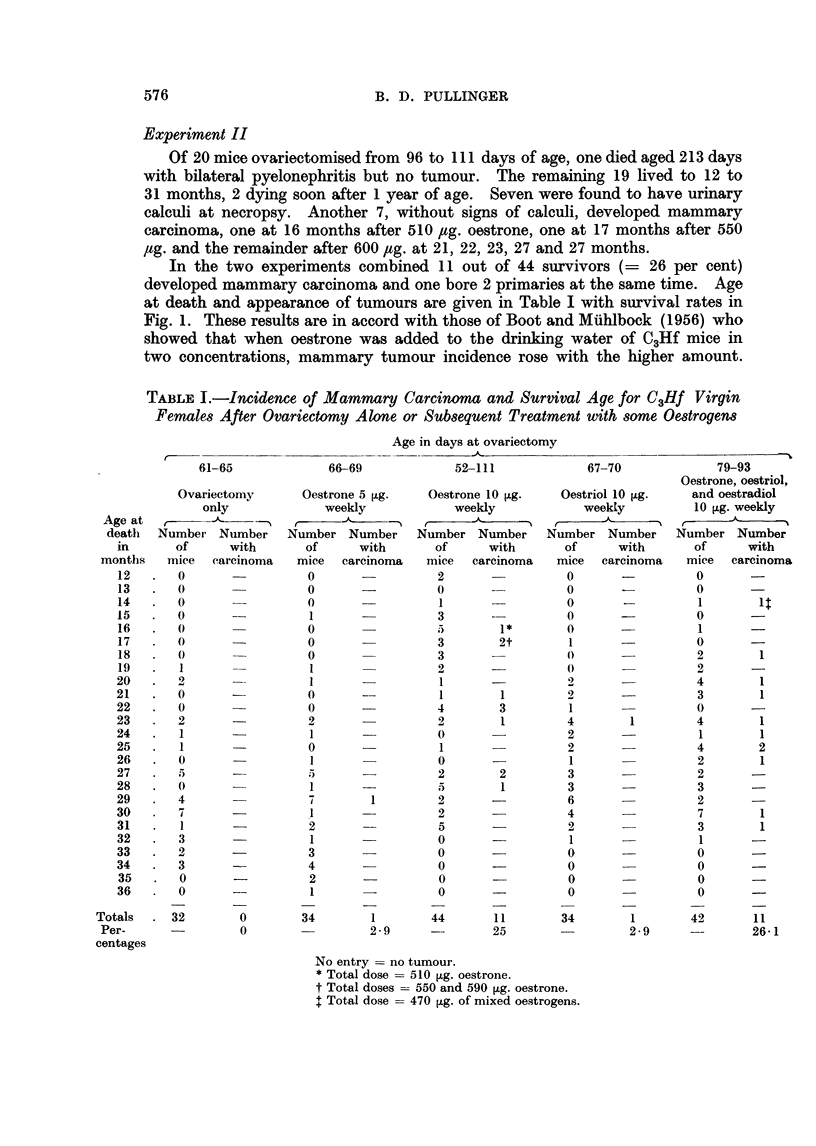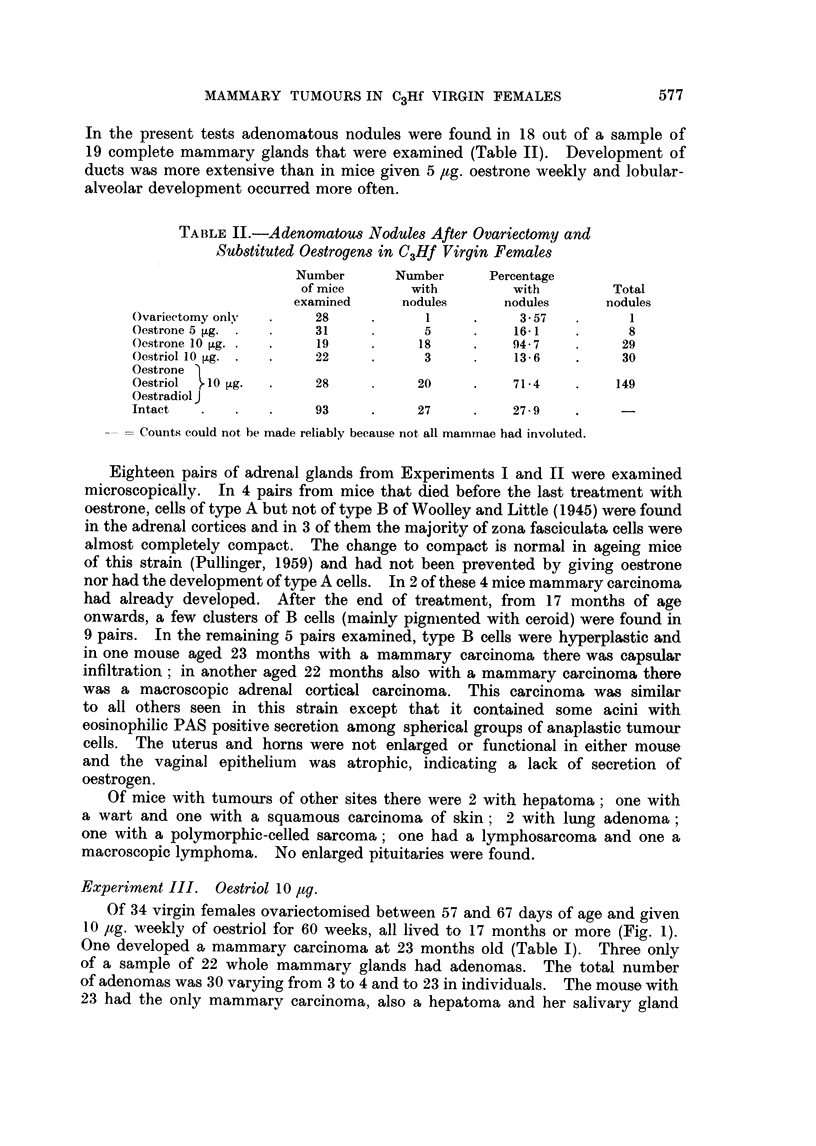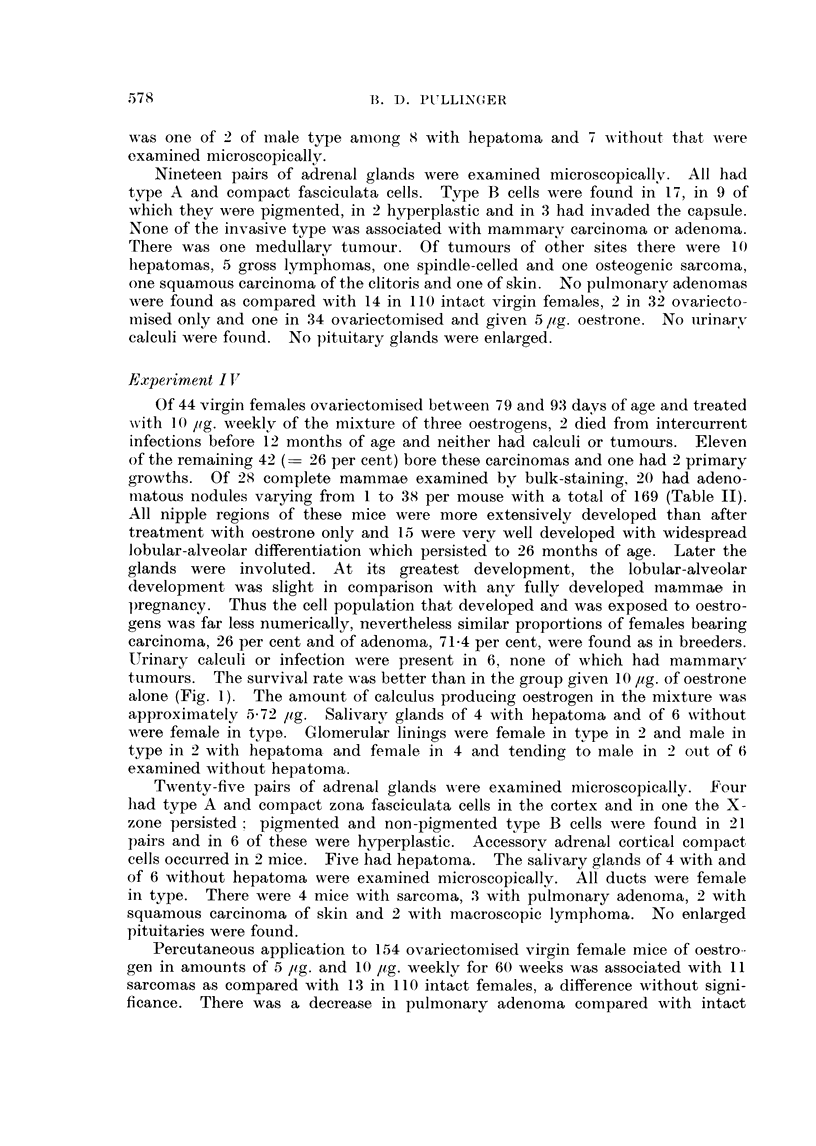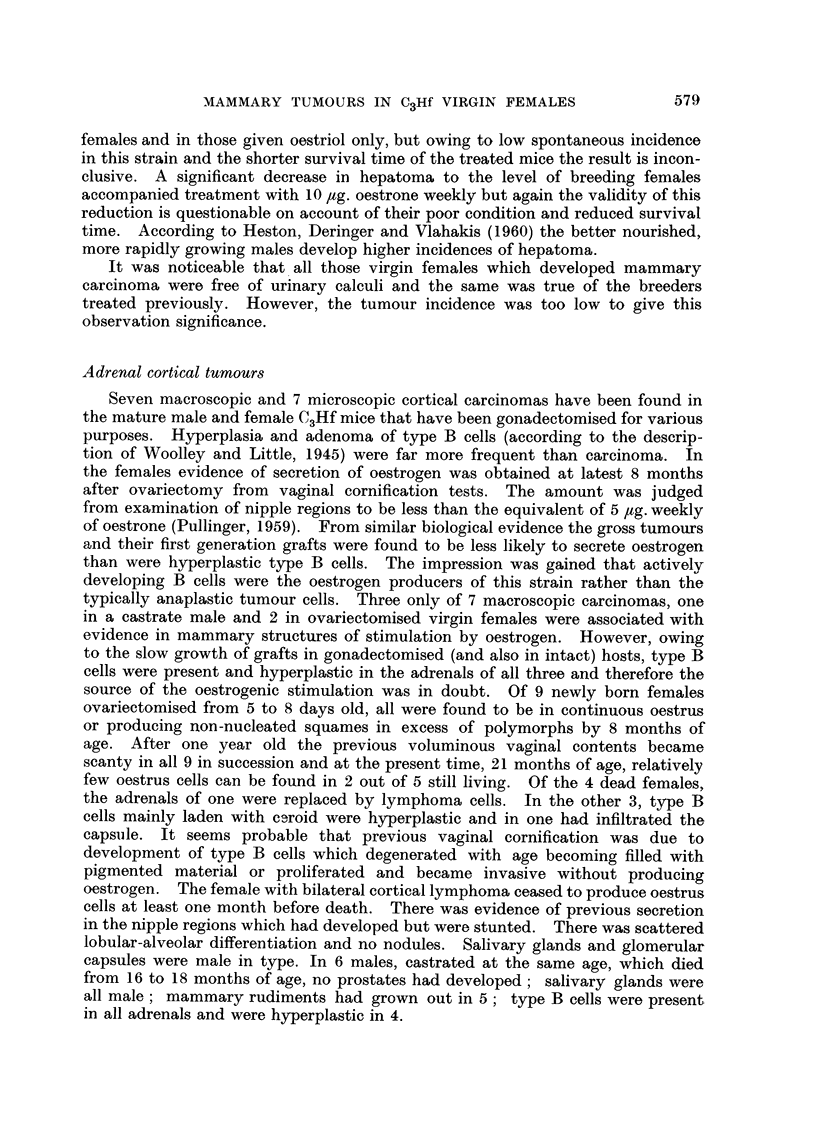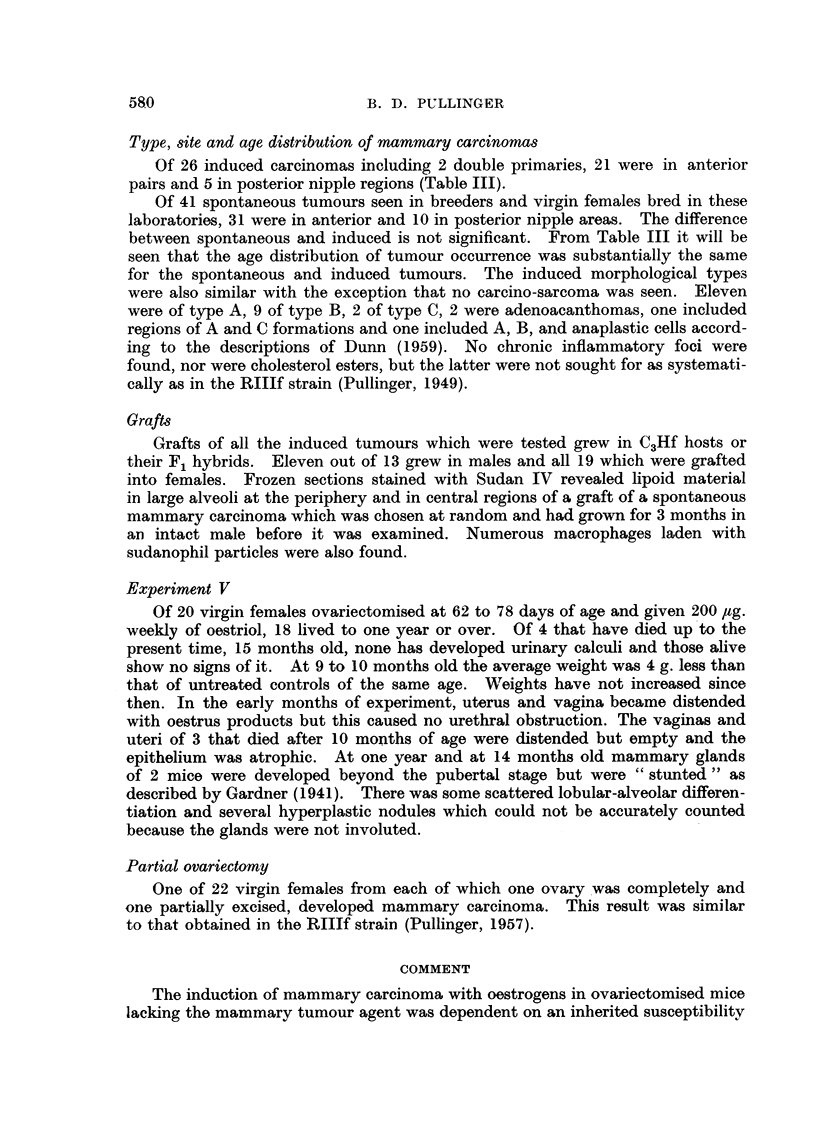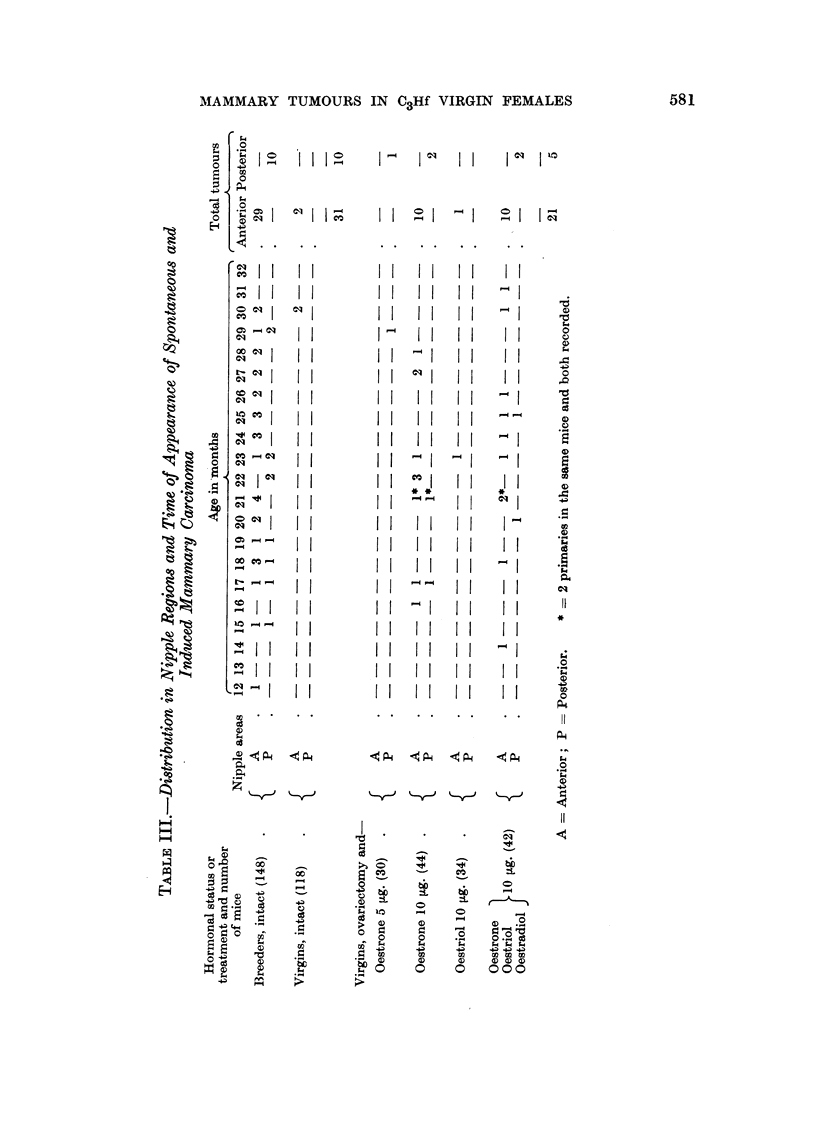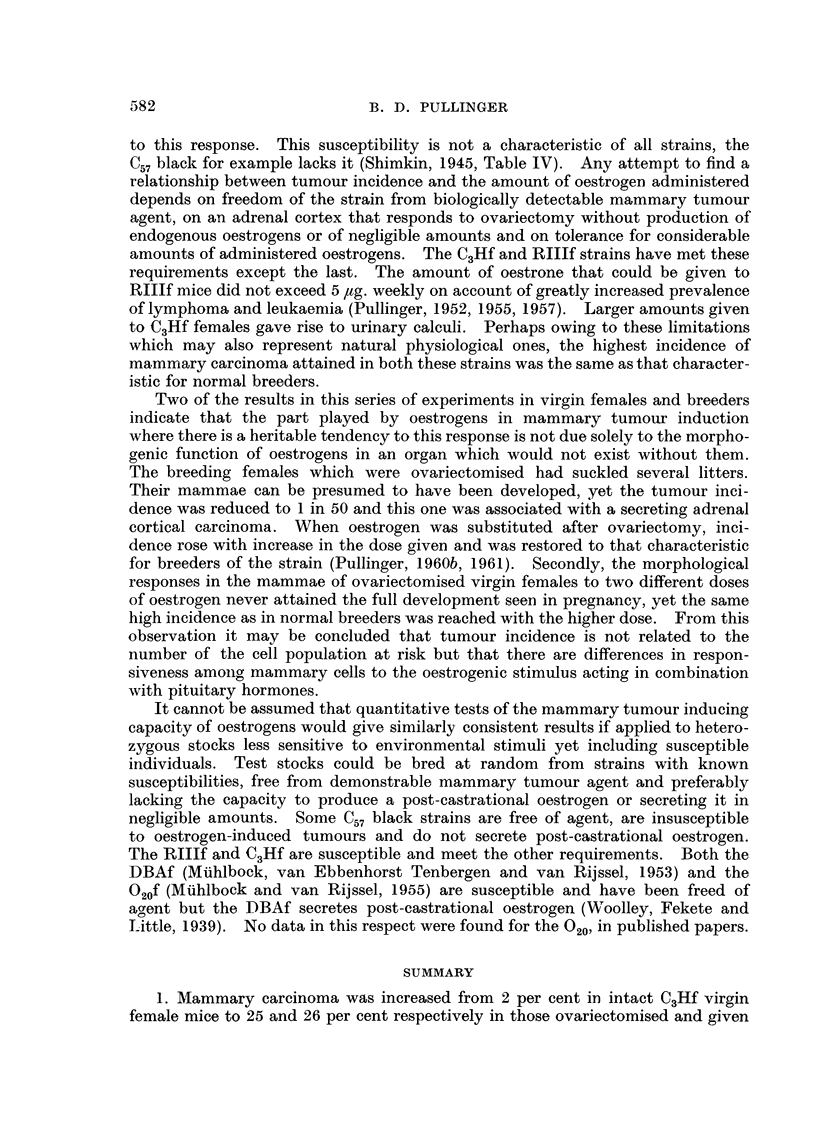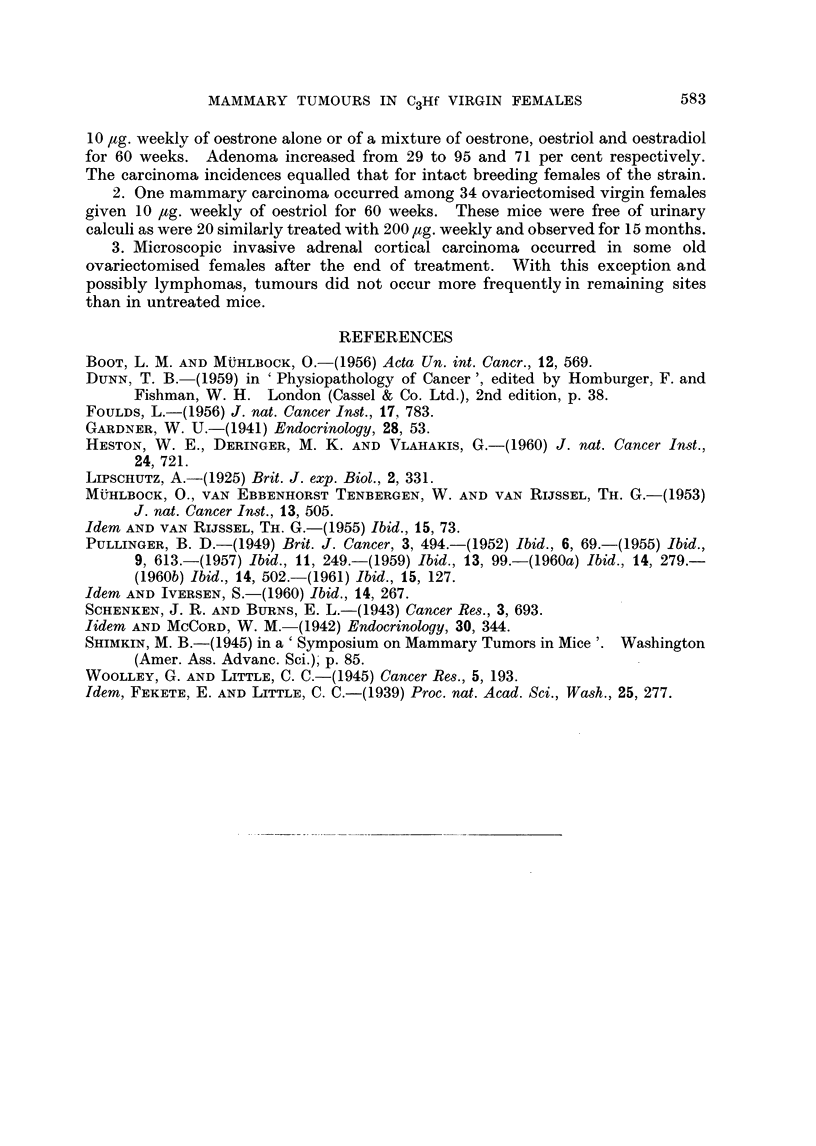# Increase in Mammary Carcinoma and Adenoma and Incidences of Other Tumours in C3Hf Virgin Females After Ovariectomy and High Dosage With Some Oestrogens

**DOI:** 10.1038/bjc.1961.67

**Published:** 1961-09

**Authors:** B. D. Pullinger


### INCREASE IN MAMMARY CARCINOMA AND ADENOMA AND INCIDENCES OF OTHER TUMOURS IN $C_3$Hf VIRGIN FEMALES AFTER OVARIECTOMY AND HIGH DOSAGE WITH SOME OESTROGENS

B. D. PULLINGER

*From the Cancer Research Department, Royal Beatson Memorial Hospital, Glasgow**

Received for publication July 20, 1961

INCIDENCES of mammary carcinoma and adenoma in $C_3$Hf virgin females, in those ovariectomised only or given 5 $\mu$g. oestrone weekly have been reported (Pullinger, 1959). The present paper records increases in carcinoma and adenoma after ovariectomy and treatment with 10 $\mu$g. weekly of oestrone alone and with 10 $\mu$g. weekly of a mixture of oestrone, oestradiol-17 $\beta$ and oestriol. Treatment with 10 $\mu$g. oestriol alone gave the same yield of mammary carcinoma as 5 $\mu$g. oestrone, namely 2·9 per cent. Tumours that arose in other sites, effects in adrenal glands, results of grafts and the site and age distribution of the mammary carcinomas are included.

### METHODS AND MATERIALS

Surplus females of the $C_3$Hf pure line colony bred for experiment were separated from males and collected in groups at weaning. The origin of the strain, management, methods of treatment and examination have been described (Pullinger and Iversen, 1960; Pullinger, 1960*a*, 1960*b*, 1961). Bilateral ovariectomies were done at 52 to 98 days of age in Experiment I and at 96 to 111 days in Experiment II. The second group was begun with older mice because deaths from urinary calculi and their consequences had been high in the first group in comparison with older and bigger breeding females (Pullinger, 1961). After ovariectomy, weekly treatment with 10 $\mu$g. oestrone dissolved in acetone was begun and continued for 60 weeks. A further test which had already been started treating virgin females with 20 $\mu$g. oestrone was abandoned on account of their poor condition and high death rate due to urinary calculi. This tendency to calculi in $C_3$H mice given oestradiol benzoate by subcutaneous injection was reported earlier by Schenken, Burns and McCord (1942), Schenken and Burns (1943).

In Experiment III, 10 $\mu$g. weekly of oestriol only was given and in Experiment IV a mixture of 10 $\mu$g. weekly of oestrone, 3 parts, oestriol, 3 parts, and oestradiol-17$\beta$, 1 part. When it became evident that no urinary calculi were formed when oestriol only was used, a test, in Experiment V, of 200 $\mu$g. weekly was begun for comparison of tolerance and tumour induction with other oestrogens.

Grafts from 6 of the mammary carcinomas induced in females and from 4 induced with oestrogen in castrated males in another experiment were made in

* Present address : Driemanskap, Private Bag, Glenroy, Transvaal, South Africa.

$C_3$Hf hosts or their $F_1$ hybrids bred by mating with $C_{57}$ black males. Some doubt was expressed by Foulds (1956) whether secretion of a milk-like substance can occur in mammary tumours in the absence of recent oestrogenic stimulation. One grafted spontaneous mammary tumour that had been growing in an intact male for 3 months was examined for lipoid secretion. Results are recorded later.

Partial ovariectomies were done on 22 virgin female mice by Lipschutz's method (1925). Neonatal gonadectomy was done on 9 female and 14 male mice from 5 to 8 days after birth in an endeavour in the first place to obtain an oestrogen secreting adrenal cortical tumour. Gonads were removed under combined bromethol (0·03 to 0·05 ml. of a 1·5 per cent suspension given intraperitoneally) and open ether anaesthesia.

### RESULTS

*Experiment I*

Of 30 females ovariectomised between 52 and 98 days of age and treated subsequently with 10 $\mu$g. oestrone weekly, one was lost and 4 died without tumour before 12 months of age. One died at 292 days from ascending pyelonephritis.

FIG. 1

| | |
|---|---|
| ○———○ | Ovariectomy only. |
| ×- - - -× | Ovariectomy and 5 μg. oestrone. |
| ○- - - -○ | Ovariectomy and 10 μg. oestrone. |
| ●———● | Ovariectomy and 10 μg. oestriol. |
| ×———× | Ovariectomy and 10 μg. {oestrone, oestriol, oestradiol.} |

Twenty-five survived of which 10 were found to have signs of retention or pyelonephritis due to urinary calculi of calcium, ammonium and magnesium phosphates. In comparison with virgin females treated previously with 5 $\mu$g. oestrone weekly (Pullinger, 1959) their death rate was increased (Fig. 1). They were poorly and under-weight during the treatment period even in the absence of calculi and infection. Four developed mammary carcinoma, one after 590 $\mu$g at 17 months of age, 2 at 22 months and 1 at 28 months. None of these 4 had signs of calculi.

*Experiment II*

Of 20 mice ovariectomised from 96 to 111 days of age, one died aged 213 days with bilateral pyelonephritis but no tumour. The remaining 19 lived to 12 to 31 months, 2 dying soon after 1 year of age. Seven were found to have urinary calculi at necropsy. Another 7, without signs of calculi, developed mammary carcinoma, one at 16 months after 510 $\mu$g. oestrone, one at 17 months after 550 $\mu$g. and the remainder after 600 $\mu$g. at 21, 22, 23, 27 and 27 months.

In the two experiments combined 11 out of 44 survivors (= 26 per cent) developed mammary carcinoma and one bore 2 primaries at the same time. Age at death and appearance of tumours are given in Table I with survival rates in Fig. 1. These results are in accord with those of Boot and Mühlbock (1956) who showed that when oestrone was added to the drinking water of $C_3$Hf mice in two concentrations, mammary tumour incidence rose with the higher amount.

TABLE I.—*Incidence of Mammary Carcinoma and Survival Age for $C_3$Hf Virgin Females After Ovariectomy Alone or Subsequent Treatment with some Oestrogens*

| | Age in days at ovariectomy | | | | | | | | | |
|---|---|---|---|---|---|---|---|---|---|---|
| | 61–65 | | 66–69 | | 52–111 | | 67–70 | | 79–93 | |
| | Ovariectomy only | | Oestrone 5 $\mu$g. weekly | | Oestrone 10 $\mu$g. weekly | | Oestriol 10 $\mu$g. weekly | | Oestrone, oestriol, and oestradiol 10 $\mu$g. weekly | |
| Age at death in months | Number of mice | Number with carcinoma | Number of mice | Number with carcinoma | Number of mice | Number with carcinoma | Number of mice | Number with carcinoma | Number of mice | Number with carcinoma |
| 12 | 0 | — | 0 | — | 2 | — | 0 | — | 0 | — |
| 13 | 0 | — | 0 | — | 0 | — | 0 | — | 0 | — |
| 14 | 0 | — | 0 | — | 1 | — | 0 | — | 1 | 1‡ |
| 15 | 0 | — | 1 | — | 3 | — | 0 | — | 0 | — |
| 16 | 0 | — | 0 | — | 5 | 1* | 0 | — | 1 | — |
| 17 | 0 | — | 0 | — | 3 | 2† | 1 | — | 0 | — |
| 18 | 0 | — | 0 | — | 3 | — | 0 | — | 2 | 1 |
| 19 | 1 | — | 1 | — | 2 | — | 0 | — | 2 | — |
| 20 | 2 | — | 1 | — | 1 | — | 2 | — | 4 | 1 |
| 21 | 0 | — | 0 | — | 1 | 1 | 2 | — | 3 | 1 |
| 22 | 0 | — | 0 | — | 4 | 3 | 1 | — | 0 | — |
| 23 | 2 | — | 2 | — | 2 | 1 | 4 | 1 | 4 | 1 |
| 24 | 1 | — | 1 | — | 0 | — | 2 | — | 1 | 1 |
| 25 | 1 | — | 0 | — | 1 | — | 2 | — | 4 | 2 |
| 26 | 0 | — | 1 | — | 0 | — | 1 | — | 2 | 1 |
| 27 | 5 | — | 5 | — | 2 | 2 | 3 | — | 2 | — |
| 28 | 0 | — | 1 | — | 5 | 1 | 3 | — | 3 | — |
| 29 | 4 | — | 7 | 1 | 2 | — | 6 | — | 2 | — |
| 30 | 7 | — | 1 | — | 2 | — | 4 | — | 7 | 1 |
| 31 | 1 | — | 2 | — | 5 | — | 2 | — | 3 | 1 |
| 32 | 3 | — | 1 | — | 0 | — | 1 | — | 1 | — |
| 33 | 2 | — | 3 | — | 0 | — | 0 | — | 0 | — |
| 34 | 3 | — | 4 | — | 0 | — | 0 | — | 0 | — |
| 35 | 0 | — | 2 | — | 0 | — | 0 | — | 0 | — |
| 36 | 0 | — | 1 | — | 0 | — | 0 | — | 0 | — |
| Totals | 32 | 0 | 34 | 1 | 44 | 11 | 34 | 1 | 42 | 11 |
| Percentages | — | 0 | — | 2·9 | — | 25 | — | 2·9 | — | 26·1 |

No entry = no tumour.

* Total dose = 510 $\mu$g. oestrone.

† Total doses = 550 and 590 $\mu$g. oestrone.

‡ Total dose = 470 $\mu$g. of mixed oestrogens.

In the present tests adenomatous nodules were found in 18 out of a sample of 19 complete mammary glands that were examined (Table II). Development of ducts was more extensive than in mice given 5 $\mu g$. oestrone weekly and lobular-alveolar development occurred more often.

TABLE II.—*Adenomatous Nodules After Ovariectomy and Substituted Oestrogens in $C_3Hf$ Virgin Females*

| | Number of mice examined | Number with nodules | Percentage with nodules | Total nodules |
|---|---|---|---|---|
| Ovariectomy only | 28 | 1 | 3·57 | 1 |
| Oestrone 5 µg. | 31 | 5 | 16·1 | 8 |
| Oestrone 10 µg. | 19 | 18 | 94·7 | 29 |
| Oestriol 10 µg. | 22 | 3 | 13·6 | 30 |
| Oestrone, Oestriol, Oestradiol } 10 µg. | 28 | 20 | 71·4 | 149 |
| Intact | 93 | 27 | 27·9 | — |

— = Counts could not be made reliably because not all mammae had involuted.

Eighteen pairs of adrenal glands from Experiments I and II were examined microscopically. In 4 pairs from mice that died before the last treatment with oestrone, cells of type A but not of type B of Woolley and Little (1945) were found in the adrenal cortices and in 3 of them the majority of zona fasciculata cells were almost completely compact. The change to compact is normal in ageing mice of this strain (Pullinger, 1959) and had not been prevented by giving oestrone nor had the development of type A cells. In 2 of these 4 mice mammary carcinoma had already developed. After the end of treatment, from 17 months of age onwards, a few clusters of B cells (mainly pigmented with ceroid) were found in 9 pairs. In the remaining 5 pairs examined, type B cells were hyperplastic and in one mouse aged 23 months with a mammary carcinoma there was capsular infiltration ; in another aged 22 months also with a mammary carcinoma there was a macroscopic adrenal cortical carcinoma. This carcinoma was similar to all others seen in this strain except that it contained some acini with eosinophilic PAS positive secretion among spherical groups of anaplastic tumour cells. The uterus and horns were not enlarged or functional in either mouse and the vaginal epithelium was atrophic, indicating a lack of secretion of oestrogen.

Of mice with tumours of other sites there were 2 with hepatoma ; one with a wart and one with a squamous carcinoma of skin ; 2 with lung adenoma ; one with a polymorphic-celled sarcoma ; one had a lymphosarcoma and one a macroscopic lymphoma. No enlarged pituitaries were found.

*Experiment III. Oestriol* 10 $\mu g$.

Of 34 virgin females ovariectomised between 57 and 67 days of age and given 10 $\mu g$. weekly of oestriol for 60 weeks, all lived to 17 months or more (Fig. 1). One developed a mammary carcinoma at 23 months old (Table I). Three only of a sample of 22 whole mammary glands had adenomas. The total number of adenomas was 30 varying from 3 to 4 and to 23 in individuals. The mouse with 23 had the only mammary carcinoma, also a hepatoma and her salivary gland was one of 2 of male type among 8 with hepatoma and 7 without that were examined microscopically.

Nineteen pairs of adrenal glands were examined microscopically. All had type A and compact fasciculata cells. Type B cells were found in 17, in 9 of which they were pigmented, in 2 hyperplastic and in 3 had invaded the capsule. None of the invasive type was associated with mammary carcinoma or adenoma. There was one medullary tumour. Of tumours of other sites there were 10 hepatomas, 5 gross lymphomas, one spindle-celled and one osteogenic sarcoma, one squamous carcinoma of the clitoris and one of skin. No pulmonary adenomas were found as compared with 14 in 110 intact virgin females, 2 in 32 ovariectomised only and one in 34 ovariectomised and given 5 $\mu$g. oestrone. No urinary calculi were found. No pituitary glands were enlarged.

*Experiment IV*

Of 44 virgin females ovariectomised between 79 and 93 days of age and treated with 10 $\mu$g. weekly of the mixture of three oestrogens, 2 died from intercurrent infections before 12 months of age and neither had calculi or tumours. Eleven of the remaining 42 (= 26 per cent) bore these carcinomas and one had 2 primary growths. Of 28 complete mammae examined by bulk-staining, 20 had adenomatous nodules varying from 1 to 38 per mouse with a total of 169 (Table II). All nipple regions of these mice were more extensively developed than after treatment with oestrone only and 15 were very well developed with widespread lobular-alveolar differentiation which persisted to 26 months of age. Later the glands were involuted. At its greatest development, the lobular-alveolar development was slight in comparison with any fully developed mammae in pregnancy. Thus the cell population that developed and was exposed to oestrogens was far less numerically, nevertheless similar proportions of females bearing carcinoma, 26 per cent and of adenoma, 71·4 per cent, were found as in breeders. Urinary calculi or infection were present in 6, none of which had mammary tumours. The survival rate was better than in the group given 10 $\mu$g. of oestrone alone (Fig. 1). The amount of calculus producing oestrogen in the mixture was approximately 5·72 $\mu$g. Salivary glands of 4 with hepatoma and of 6 without were female in type. Glomerular linings were female in type in 2 and male in type in 2 with hepatoma and female in 4 and tending to male in 2 out of 6 examined without hepatoma.

Twenty-five pairs of adrenal glands were examined microscopically. Four had type A and compact zona fasciculata cells in the cortex and in one the X-zone persisted ; pigmented and non-pigmented type B cells were found in 21 pairs and in 6 of these were hyperplastic. Accessory adrenal cortical compact cells occurred in 2 mice. Five had hepatoma. The salivary glands of 4 with and of 6 without hepatoma were examined microscopically. All ducts were female in type. There were 4 mice with sarcoma, 3 with pulmonary adenoma, 2 with squamous carcinoma of skin and 2 with macroscopic lymphoma. No enlarged pituitaries were found.

Percutaneous application to 154 ovariectomised virgin female mice of oestrogen in amounts of 5 $\mu$g. and 10 $\mu$g. weekly for 60 weeks was associated with 11 sarcomas as compared with 13 in 110 intact females, a difference without significance. There was a decrease in pulmonary adenoma compared with intact females and in those given oestriol only, but owing to low spontaneous incidence in this strain and the shorter survival time of the treated mice the result is inconclusive. A significant decrease in hepatoma to the level of breeding females accompanied treatment with 10 $\mu$g. oestrone weekly but again the validity of this reduction is questionable on account of their poor condition and reduced survival time. According to Heston, Deringer and Vlahakis (1960) the better nourished, more rapidly growing males develop higher incidences of hepatoma.

It was noticeable that all those virgin females which developed mammary carcinoma were free of urinary calculi and the same was true of the breeders treated previously. However, the tumour incidence was too low to give this observation significance.

*Adrenal cortical tumours*

Seven macroscopic and 7 microscopic cortical carcinomas have been found in the mature male and female $C_3Hf$ mice that have been gonadectomised for various purposes. Hyperplasia and adenoma of type B cells (according to the description of Woolley and Little, 1945) were far more frequent than carcinoma. In the females evidence of secretion of oestrogen was obtained at latest 8 months after ovariectomy from vaginal cornification tests. The amount was judged from examination of nipple regions to be less than the equivalent of 5 $\mu$g. weekly of oestrone (Pullinger, 1959). From similar biological evidence the gross tumours and their first generation grafts were found to be less likely to secrete oestrogen than were hyperplastic type B cells. The impression was gained that actively developing B cells were the oestrogen producers of this strain rather than the typically anaplastic tumour cells. Three only of 7 macroscopic carcinomas, one in a castrate male and 2 in ovariectomised virgin females were associated with evidence in mammary structures of stimulation by oestrogen. However, owing to the slow growth of grafts in gonadectomised (and also in intact) hosts, type B cells were present and hyperplastic in the adrenals of all three and therefore the source of the oestrogenic stimulation was in doubt. Of 9 newly born females ovariectomised from 5 to 8 days old, all were found to be in continuous oestrus or producing non-nucleated squames in excess of polymorphs by 8 months of age. After one year old the previous voluminous vaginal contents became scanty in all 9 in succession and at the present time, 21 months of age, relatively few oestrus cells can be found in 2 out of 5 still living. Of the 4 dead females, the adrenals of one were replaced by lymphoma cells. In the other 3, type B cells mainly laden with ceroid were hyperplastic and in one had infiltrated the capsule. It seems probable that previous vaginal cornification was due to development of type B cells which degenerated with age becoming filled with pigmented material or proliferated and became invasive without producing oestrogen. The female with bilateral cortical lymphoma ceased to produce oestrus cells at least one month before death. There was evidence of previous secretion in the nipple regions which had developed but were stunted. There was scattered lobular-alveolar differentiation and no nodules. Salivary glands and glomerular capsules were male in type. In 6 males, castrated at the same age, which died from 16 to 18 months of age, no prostates had developed ; salivary glands were all male ; mammary rudiments had grown out in 5 ; type B cells were present in all adrenals and were hyperplastic in 4.

*Type, site and age distribution of mammary carcinomas*

Of 26 induced carcinomas including 2 double primaries, 21 were in anterior pairs and 5 in posterior nipple regions (Table III).

Of 41 spontaneous tumours seen in breeders and virgin females bred in these laboratories, 31 were in anterior and 10 in posterior nipple areas. The difference between spontaneous and induced is not significant. From Table III it will be seen that the age distribution of tumour occurrence was substantially the same for the spontaneous and induced tumours. The induced morphological types were also similar with the exception that no carcino-sarcoma was seen. Eleven were of type A, 9 of type B, 2 of type C, 2 were adenoacanthomas, one included regions of A and C formations and one included A, B, and anaplastic cells according to the descriptions of Dunn (1959). No chronic inflammatory foci were found, nor were cholesterol esters, but the latter were not sought for as systematically as in the RIIIf strain (Pullinger, 1949).

*Grafts*

Grafts of all the induced tumours which were tested grew in $C_3$Hf hosts or their $F_1$ hybrids. Eleven out of 13 grew in males and all 19 which were grafted into females. Frozen sections stained with Sudan IV revealed lipoid material in large alveoli at the periphery and in central regions of a graft of a spontaneous mammary carcinoma which was chosen at random and had grown for 3 months in an intact male before it was examined. Numerous macrophages laden with sudanophil particles were also found.

*Experiment V*

Of 20 virgin females ovariectomised at 62 to 78 days of age and given 200 $\mu$g. weekly of oestriol, 18 lived to one year or over. Of 4 that have died up to the present time, 15 months old, none has developed urinary calculi and those alive show no signs of it. At 9 to 10 months old the average weight was 4 g. less than that of untreated controls of the same age. Weights have not increased since then. In the early months of experiment, uterus and vagina became distended with oestrus products but this caused no urethral obstruction. The vaginas and uteri of 3 that died after 10 months of age were distended but empty and the epithelium was atrophic. At one year and at 14 months old mammary glands of 2 mice were developed beyond the pubertal stage but were "stunted" as described by Gardner (1941). There was some scattered lobular-alveolar differentiation and several hyperplastic nodules which could not be accurately counted because the glands were not involuted.

*Partial ovariectomy*

One of 22 virgin females from each of which one ovary was completely and one partially excised, developed mammary carcinoma. This result was similar to that obtained in the RIIIf strain (Pullinger, 1957).

### COMMENT

The induction of mammary carcinoma with oestrogens in ovariectomised mice lacking the mammary tumour agent was dependent on an inherited susceptibility

TABLE III.—*Distribution in Nipple Regions and Time of Appearance of Spontaneous and Induced Mammary Carcinoma*

| Hormonal status or treatment and number of mice | Nipple areas | Age in months: 12 | 13 | 14 | 15 | 16 | 17 | 18 | 19 | 20 | 21 | 22 | 23 | 24 | 25 | 26 | 27 | 28 | 29 | 30 | 31 | 32 | Total tumours: Anterior | Posterior |
|---|---|---|---|---|---|---|---|---|---|---|---|---|---|---|---|---|---|---|---|---|---|---|---|---|
| Breeders, intact (148) | A | 1 | — | — | 1 | — | 1 | 3 | 1 | 2 | 4 | — | 1 | 3 | 3 | 2 | 2 | 2 | 1 | 2 | — | — | 29 | — |
| | P | — | — | — | 1 | — | 1 | 1 | 1 | — | — | 2 | 2 | — | — | — | — | — | 2 | — | — | — | — | 10 |
| Virgins, intact (118) | A | — | — | — | — | — | — | — | — | — | — | — | — | — | — | — | — | — | — | 2 | — | — | 2 | — |
| | P | — | — | — | — | — | — | — | — | — | — | — | — | — | — | — | — | — | — | — | — | — | — | — |
| | | | | | | | | | | | | | | | | | | | | | | | 31 | 10 |
| Virgins, ovariectomy and— | | | | | | | | | | | | | | | | | | | | | | | | |
| Oestrone 5 μg. (30) | A | — | — | — | — | — | — | — | — | — | — | — | — | — | — | — | — | — | — | — | — | — | — | — |
| | P | — | — | — | — | — | — | — | — | — | — | — | — | — | — | — | — | — | 1 | — | — | — | — | 1 |
| Oestrone 10 μg. (44) | A | — | — | — | — | 1 | 1 | — | — | — | 1* | 3 | 1 | — | — | — | 2 | 1 | — | — | — | — | 10 | — |
| | P | — | — | — | — | — | 1 | — | — | — | 1* | — | — | — | — | — | — | — | — | — | — | — | — | 2 |
| Oestriol 10 μg. (34) | A | — | — | — | — | — | — | — | — | — | — | — | 1 | — | — | — | — | — | — | — | — | — | 1 | — |
| | P | — | — | — | — | — | — | — | — | — | — | — | — | — | — | — | — | — | — | — | — | — | — | — |
| Oestrone, Oestriol, Oestradiol 10 μg. (42) | A | — | — | 1 | — | — | — | 1 | — | — | 2* | — | 1 | 1 | 1 | 1 | — | — | — | 1 | 1 | — | 10 | — |
| | P | — | — | — | — | — | — | — | — | 1 | — | — | — | — | 1 | — | — | — | — | — | — | — | — | 2 |
| | | | | | | | | | | | | | | | | | | | | | | | 21 | 5 |

A = Anterior; P = Posterior. * = 2 primaries in the same mice and both recorded.

to this response. This susceptibility is not a characteristic of all strains, the $C_{57}$ black for example lacks it (Shimkin, 1945, Table IV). Any attempt to find a relationship between tumour incidence and the amount of oestrogen administered depends on freedom of the strain from biologically detectable mammary tumour agent, on an adrenal cortex that responds to ovariectomy without production of endogenous oestrogens or of negligible amounts and on tolerance for considerable amounts of administered oestrogens. The $C_3$Hf and RIIIf strains have met these requirements except the last. The amount of oestrone that could be given to RIIIf mice did not exceed 5 $\mu$g. weekly on account of greatly increased prevalence of lymphoma and leukaemia (Pullinger, 1952, 1955, 1957). Larger amounts given to $C_3$Hf females gave rise to urinary calculi. Perhaps owing to these limitations which may also represent natural physiological ones, the highest incidence of mammary carcinoma attained in both these strains was the same as that characteristic for normal breeders.

Two of the results in this series of experiments in virgin females and breeders indicate that the part played by oestrogens in mammary tumour induction where there is a heritable tendency to this response is not due solely to the morphogenic function of oestrogens in an organ which would not exist without them. The breeding females which were ovariectomised had suckled several litters. Their mammae can be presumed to have been developed, yet the tumour incidence was reduced to 1 in 50 and this one was associated with a secreting adrenal cortical carcinoma. When oestrogen was substituted after ovariectomy, incidence rose with increase in the dose given and was restored to that characteristic for breeders of the strain (Pullinger, 1960*b*, 1961). Secondly, the morphological responses in the mammae of ovariectomised virgin females to two different doses of oestrogen never attained the full development seen in pregnancy, yet the same high incidence as in normal breeders was reached with the higher dose. From this observation it may be concluded that tumour incidence is not related to the number of the cell population at risk but that there are differences in responsiveness among mammary cells to the oestrogenic stimulus acting in combination with pituitary hormones.

It cannot be assumed that quantitative tests of the mammary tumour inducing capacity of oestrogens would give similarly consistent results if applied to heterozygous stocks less sensitive to environmental stimuli yet including susceptible individuals. Test stocks could be bred at random from strains with known susceptibilities, free from demonstrable mammary tumour agent and preferably lacking the capacity to produce a post-castrational oestrogen or secreting it in negligible amounts. Some $C_{57}$ black strains are free of agent, are insusceptible to oestrogen-induced tumours and do not secrete post-castrational oestrogen. The RIIIf and $C_3$Hf are susceptible and meet the other requirements. Both the DBAf (Mühlbock, van Ebbenhorst Tenbergen and van Rijssel, 1953) and the $O_{20}$f (Mühlbock and van Rijssel, 1955) are susceptible and have been freed of agent but the DBAf secretes post-castrational oestrogen (Woolley, Fekete and Little, 1939). No data in this respect were found for the $O_{20}$, in published papers.

### SUMMARY

1. Mammary carcinoma was increased from 2 per cent in intact $C_3$Hf virgin female mice to 25 and 26 per cent respectively in those ovariectomised and given

10 $\mu$g. weekly of oestrone alone or of a mixture of oestrone, oestriol and oestradiol for 60 weeks. Adenoma increased from 29 to 95 and 71 per cent respectively. The carcinoma incidences equalled that for intact breeding females of the strain.

2. One mammary carcinoma occurred among 34 ovariectomised virgin females given 10 $\mu$g. weekly of oestriol for 60 weeks. These mice were free of urinary calculi as were 20 similarly treated with 200 $\mu$g. weekly and observed for 15 months.

3. Microscopic invasive adrenal cortical carcinoma occurred in some old ovariectomised females after the end of treatment. With this exception and possibly lymphomas, tumours did not occur more frequently in remaining sites than in untreated mice.

### REFERENCES

Boot, L. M. and Mühlbock, O.—(1956) *Acta Un. int. Cancr.*, **12**, 569.

Dunn, T. B.—(1959) in ' Physiopathology of Cancer ', edited by Homburger, F. and Fishman, W. H. London (Cassel & Co. Ltd.), 2nd edition, p. 38.

Foulds, L.—(1956) *J. nat. Cancer Inst.*, **17**, 783.

Gardner, W. U.—(1941) *Endocrinology*, **28**, 53.

Heston, W. E., Deringer, M. K. and Vlahakis, G.—(1960) *J. nat. Cancer Inst.*, **24**, 721.

Lipschutz, A.—(1925) *Brit. J. exp. Biol.*, **2**, 331.

Mühlbock, O., van Ebbenhorst Tenbergen, W. and van Rijssel, Th. G.—(1953) *J. nat. Cancer Inst.*, **13**, 505.

*Idem* and van Rijssel, Th. G.—(1955) *Ibid.*, **15**, 73.

Pullinger, B. D.—(1949) *Brit. J. Cancer*, **3**, 494.—(1952) *Ibid.*, **6**, 69.—(1955) *Ibid.*, **9**, 613.—(1957) *Ibid.*, **11**, 249.—(1959) *Ibid.*, **13**, 99.—(1960*a*) *Ibid.*, **14**, 279.—(1960*b*) *Ibid.*, **14**, 502.—(1961) *Ibid.*, **15**, 127.

*Idem* and Iversen, S.—(1960) *Ibid.*, **14**, 267.

Schenken, J. R. and Burns, E. L.—(1943) *Cancer Res.*, **3**, 693.

*Iidem* and McCord, W. M.—(1942) *Endocrinology*, **30**, 344.

Shimkin, M. B.—(1945) in a ' Symposium on Mammary Tumors in Mice '. Washington (Amer. Ass. Advanc. Sci.), p. 85.

Woolley, G. and Little, C. C.—(1945) *Cancer Res.*, **5**, 193.

*Idem*, Fekete, E. and Little, C. C.—(1939) *Proc. nat. Acad. Sci., Wash.*, **25**, 277.